# Vaccine efficacy and T helper cell differentiation change with aging

**DOI:** 10.18632/oncotarget.9254

**Published:** 2016-05-09

**Authors:** Julie S. Lefebvre, Erica C. Lorenzo, April R. Masters, Jacob W. Hopkins, Sheri M. Eaton, Stephen T. Smiley, Laura Haynes

**Affiliations:** ^1^ The Trudeau Institute, Saranac Lake, NY, United States of America; ^2^ Department of Immunology and Center on Aging, University of Connecticut Health Center, Farmington, CT, United States of America; ^3^ Département de Pneumologie, Centre de Recherche de l'Institut Universitaire de Cardiologie et Pneumologie de Québec, Québec, QC, Canada; ^4^ NIAID/NIH, Bethesda, MD, USA

**Keywords:** aging, influenza, Th subsets, influenza vaccination, antibody, Gerotarget

## Abstract

Influenza and pneumonia are leading causes of death in elderly populations. With age, there is an increased inflammatory response and slower viral clearance during influenza infection which increases the risk of extended illness and mortality. Here we employ a preclinical murine model of influenza infection to examine the protective capacity of vaccination with influenza nucleoprotein (NP). While NP vaccination reduces influenza-induced lung inflammation in young mice, aged mice do not show this reduction, but are protected from influenza-induced mortality. Aged mice do make a significant amount of NP-specific IgG and adoptive transfer experiments show that NP antibody can protect from death but cannot reduce lung inflammation. Furthermore, young but not aged vaccinated mice generate significant numbers of NP-specific T cells following subsequent infection and few of these T cells are found in aged lungs early during infection. Importantly, aged CD4 T cells have a propensity to differentiate towards a T follicular helper (Tfh) phenotype rather than a T helper 1 (Th1) phenotype that predominates in the young. Since Th1 cells are important in viral clearance, reduced Th1 differentiation in the aged is critical and could account for some or all of the age-related differences in vaccine responses and infection resolution.

## INTRODUCTION

Through decades of research, it has become clear that the process of aging impacts the function of most bodily systems. One of the major concerns with age-associated declines is the increased susceptibility to infections. Influenza infection is one of the top ten killers of elderly people in the world, with the oldest being most at risk [1]. Furthermore, aging impacts many facets of the immune system. Cells of the innate [2-6] and adaptive [7-11] arms exhibit altered phenotypes, along with diminished and dysregulated responses, as demonstrated in both human and mouse studies. A complex network of communication is required between the innate and adaptive cell types to generate a robust immune response to influenza vaccination and protection from infection. The implications of age related changes in these cell types impacting the efficacy of vaccination, however, are not well defined.

Studies in aged mice have demonstrated that clearance of influenza virus is delayed and T cell responses are reduced when compared to young mice, a situation that closely resembles human influenza infection in the elderly [12-14]. Lingering virus in aged lungs was also associated with increased numbers of innate cells, such as granulocytes, macrophages and dendritic cells [15]. In addition, inflammatory cytokines, such as IL-1 and IL-6 peak later and stay elevated longer in lung homogenates from aged influenza infected mice [15]. This indicates that the inability of aged individuals to efficiently clear virus is associated with extended inflammation in the lungs. Lung inflammation has also been correlated with increased susceptibility to secondary bacterial infections [16-18], which not only can result in bacterial pneumonia but can also lead to bacterial dissemination and sepsis, which are especially serious for elderly individuals. One of the most effective ways to prevent influenza infection and reduce secondary bacterial infections is through the administration of an influenza vaccine [19-21]. Importantly, we have shown that prior immunity to influenza, such as that generated by vaccination with influenza nucleoprotein (NP), significantly reduced lung inflammation and damage. This reduction in lung inflammation was also correlated with decreased susceptibility to secondary bacterial infection and subsequent death [18, 22].

Inactivated and recombinant influenza vaccines induce a robust antibody and CD4 T cell response. Neutralizing antibodies, such as those directed against hemaglutinin, work to prevent infection, while non-neutralizing antibodies, such as those against NP can facilitate viral clearance *via* mechanisms such as antibody-dependent cell-mediated cytotoxicity [23]. Responding CD4 T cells differentiate into T follicular helper (Tfh) cells, which are required for germinal center formation and a high affinity antibody response. They also differentiate into type 1 helper (Th1) cells, which are important for killing virally infected targets in the lungs [24]. Since these influenza-specific CD4 T cells are primed during vaccination, they are poised to traffic to the lungs at the first sign of infection, prior to other unprimed T cell populations. This provides a distinct head start on fighting the virus in vaccinated individuals, resulting in quicker clearance and less severe illness. This orchestrated differentiation of CD4 T cell subsets during an immune response is controlled by currently unknown mechanisms but it is vital that it occur correctly in order for proper protective immunity to develop.

In this report, we examine the efficacy of an influenza NP vaccine in a preclinical model of influenza infection in the elderly. Our results show that NP vaccination of young mice leads to reduced inflammatory mediators in the lungs, high titers of NP-specific antibody and expansion of NP-specific CD4 T cells in the spleen and lungs. In contrast, vaccination of aged mice induces enough of an antibody response to protect from influenza-induced death, but vaccination does not protect from lung inflammation, possibly due to reduced Th1 differentiation and homing to infected lungs. Thus, our results point to a novel finding that the two mechanisms of vaccine-mediated protection, antibody and lung-homing T cell effector generation, seem to be differentially affected by aging.

## RESULTS

### Aged mice display increased lung damage and lingering inflammation after influenza infection

**Figure 1:**
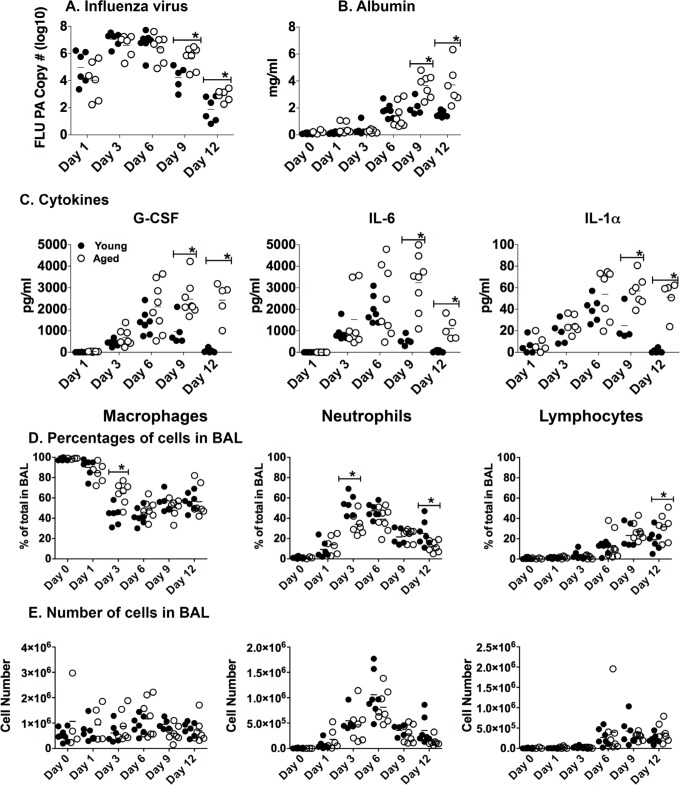
Lingering inflammatory cytokines and lung damage in aged lungs during influenza infection Young (2-3 mos.) and aged (19-22 mos.) C57BL/6 mice were infected intranasally with 400 EID_50_ of PR8 influenza. On 1, 3, 6, 9 and 12 dpi, BAL fluid and cells and lung tissue was harvested; mRNA was isolated from lung tissue. **A.** The total number of copies of influenza PA was determined by real-time PCR of mRNA from lung tissue. **B.** Albumin in the BAL was determined by ELISA and **C.** Cytokines in the BAL were determined by multiplex analysis. The percentage **D.** and number **E.** of macrophages, neutrophils and lymphocytes in the BAL was determined by differential staining. For all experiments, data shown is combined from 2 independent experiments and each symbol represents a single animal; line shows the mean. *p < 0.05 by 2 way ANOVA with Bonferroni posthoc correction.

In order to investigate the impact aging has on the kinetics of the immune response during influenza infection, young and aged C57BL/6 mice were intranasally infected with PR8 influenza. Lung tissue and bronchiolar lavage (BAL) fluid and cells were harvested at various time points up to 12 days post-infection (dpi). Both young and aged groups reached a similar peak of viral load between days 3 to 6 of infection. Virus levels in both groups were decreasing by day 9 but the aged groups still exhibited significantly higher copy numbers at 9 and 12 dpi (Figure 1A). While this age-related difference appears subtle at these later time points, the data are expressed in log_10_ copy numbers of the influenza acid polymerase (PA) gene [25]. Thus, the differences between the young and aged groups are substantial and the aged mice have delayed clearance of influenza virus in the lung tissue.

The delay of viral clearance in the aged mice could also cause increased lung damage and inflammation. Quantifying albumin levels BAL is a good measure of lung damage [26], thus we examined this during the course of infection (Figure 1B). Interestingly, young mice had slightly elevated levels of albumin in the BAL, which plateaued between 6 and 12 dpi. Aged mice also had elevated levels of albumin, which continued to increase from 6 to 12 dpi in comparison to the young mice at each respective time point. This indicates that there is ongoing lung damage that is not resolving in the aged lungs.

The BAL fluid was also examined for inflammatory cytokines that are critical for viral clearance (Figure 1C). We examined the levels G-CSF, which promotes the accumulation of granulocytes and facilitates viral clearance [27], IL-6, which is vitally important for neutrophil survival and viral clearance [28], and IL-1α, which mediates lung pathology while also enhancing survival *via* activation of the adaptive immune response and clearance of the virus [29]. Young mice had increased levels of inflammatory cytokines G-CSF, IL-6, and IL-1α, which peaked at 6 dpi and then returned to baseline by day 12 as the infection was resolving. Aged mice, however, had significantly higher levels of each cytokine on days 9 and 12 without a return to baseline levels that was seen with the young groups. Additional cytokines and chemokines also followed a similar pattern of being elevated in the aged groups, especially at later time points (Sup Figure 1A), while others, such as the effector cytokines IFNγ and IL-10, were delayed in the aged (Sup Figure 1B) and some chemokines important for recruiting innate and adaptive immune cells to the lungs during infection were found to be higher in the young groups (Sup Figure 1C). Taken together, these results show that along with a delay in the clearance of influenza infection and generation of effector cytokines in the aged mice, there is also significantly increased lung damage and lingering inflammatory cytokines and chemokines, possibly in response to the lingering virus in the aged lungs.

In order to determine if age-related differences in infiltrating cells could have been contributing to this dramatic increase in inflammatory cytokines and chemokines, we also examined cells in the BAL. Interestingly, there were few significant differences between young and aged groups with regards to the percentages of specific cell types in the BAL population, with slightly more neutrophils in the young and slightly more macrophages in the aged at 3 dpi (Figure 1D). Importantly, there were no significant differences in cell numbers between the young and aged groups throughout the infection (Figure 1E). Thus, it is unlikely that the ongoing inflammation seen in the aged lungs is due to excessive cellular infiltrate, but might be attributed to age-related changes in the quantities of cytokines produced per cell.

### Effect of NP vaccination aged responses to influenza

Our previous studies have shown that one of the easiest and most straightforward ways to reduce inflammatory cytokines and chemokines in the lungs during influenza infection is by prior vaccination [18, 22]. Vaccination with influenza NP is a useful approach since this recombinant protein is easy to generate and it induces robust heterosubtypic immunity [30], thereby overcoming two major deficiencies of the seasonal inactivated whole virus vaccines.

**Figure 2:**
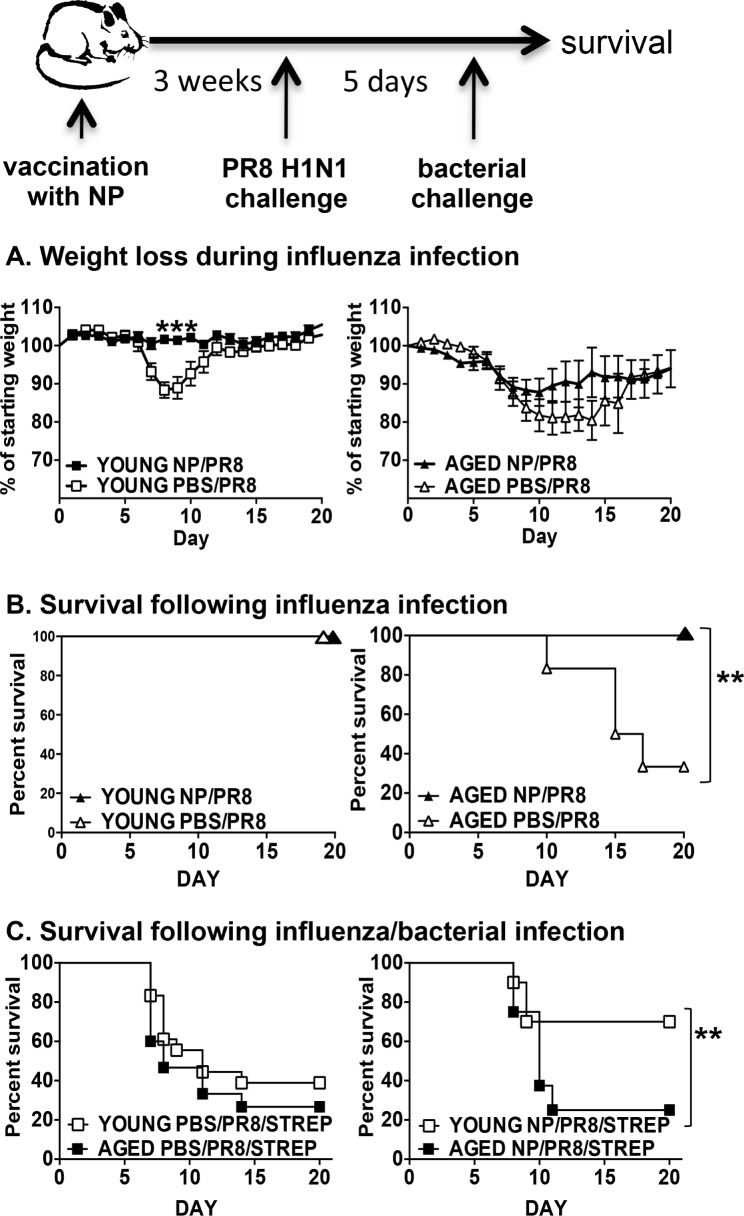
Protection from influenza and secondary bacterial infection by NP vaccination Young (2-3 mos.) and aged (19-22 mos.) C57BL/6 mice were vaccinated with NP or PBS on days −31 and -21. On day 0, they were infected intranasally with 400 EID_50_ of PR8 influenza. **A.** Weight loss and **B.** survival were monitored following infection. **C.** On day 0, NP (right graph) or PBS (left graph) vaccinated young and aged mice were infected intranasally with 400 EID_50_ of PR8 influenza and then 5 dpi, infected with 250 CFU of *Streptococcus pneumoniae* and survival was monitored. Data shown is combined from 2 independent experiments with a total of 8 to 10 mice per group. *p < 0.05 by Student's *t* test comparing vaccinated and control groups; **p < 0.05 by Log rank test.

Young and aged mice were vaccinated with recombinant NP or PBS as a control and then infected with PR8 influenza; weight loss and survival were then monitored. NP vaccination prevented weight loss in young mice following influenza infection. While vaccination had no significant protective effect on weight loss in the aged group, there was a trend towards vaccinated mice not losing as much weight as the controls (Figure 2A). Importantly, we also found that NP vaccination did confer protection from mortality in the aged cohort, with 100% survival in the vaccinated group compared to 30 percent in the PBS vaccinated aged group (Figure 2B). Thus, while vaccination of the aged mice does not protect from influenza-induced weight loss, it does rescue aged mice from death.

Another beneficial characteristic of this NP vaccination is that it protects from death due to secondary bacterial infection following influenza in young mice [18]. Thus, we next examined if the NP vaccinated aged mice could also be similarly protected. Mice were vaccinated with NP or PBS and then given an influenza infection. Five dpi, mice were given a secondary infection with a sublethal dose of *Streptococcus pneumonia* (survival graph of mice given Strep alone shown in Sup Figure 2). We chose day 5 for bacterial challenge since it is prior to any significant weight loss in influenza-infected young and aged groups (see Figure 2A). Not surprisingly, both control PBS vaccinated young and aged groups succumbed to the dual infection (Figure 2C, left graph). Young NP vaccinated mice were protected from death, as we have shown previously [18], but aged NP vaccinated mice were not protected (Figure 2C, right graph). Therefore, while NP vaccinated aged mice were rescued from influenza-induced death, they still remained susceptible to death due to secondary bacterial infection.

**Figure 3:**
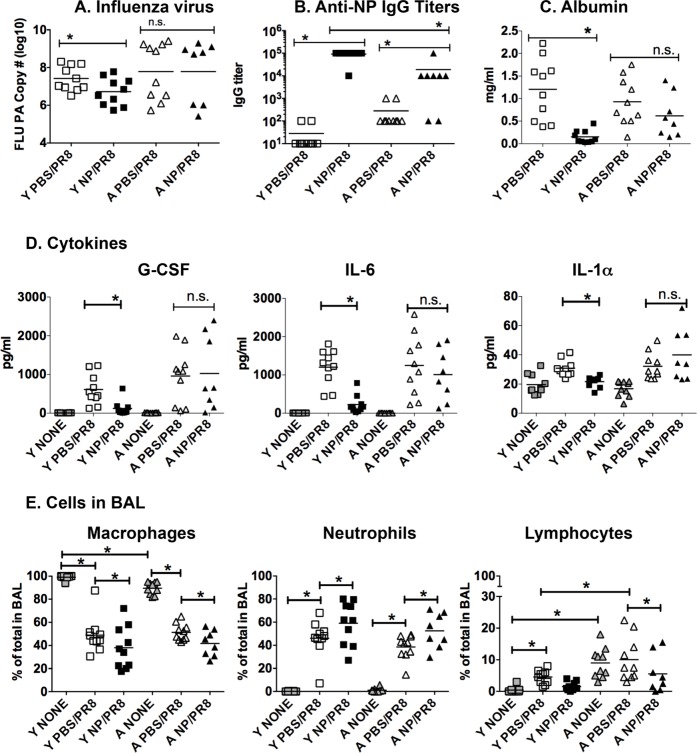
NP vaccination reduces lung inflammatory mediators and damage in influenza infected young but not aged mice Young [Y] (2-3 mos.) and aged [A] (19-22 mos.) C57BL/6 mice were vaccinated with NP or PBS on days -31 and -21. On day 0, they were infected intranasally with 400 EID_50_ of PR8 influenza. On 5 dpi, **A.** lungs were harvested and mRNA was isolated to quantitate influenza PA by real-time PCR. **B.** Anti-NP IgG titers in serum were determined by ELISA. Albumin **C.** and cytokines **D.** in the BAL were determined by ELISA and multiplex analysis, respectively. **E.** The percentage of macrophages, neutrophils and lymphocytes in the BAL was determined by differential staining. Groups labeled “none” represent BAL harvested from young or aged naive mice. Data shown is combined from 2 independent experiments and each symbol represents a single animal; line shows the mean. *p < 0.05 by 2 way ANOVA for A, B, C or 3 way ANOVA for D and E with Bonferroni posthoc correction.

We then went on to examine other parameters in these vaccinated mice. Young and aged mice vaccinated with NP or PBS were challenged with PR8 influenza and examined 5 dpi since this was the day of bacterial challenge in the previous set of experiments. Neither young nor aged NP vaccinated/infected (NP/PR8) mice had a significant reduction of virus in the lungs when compared to the PBS vaccinated control groups (PBS/PR8) (Figure 3A). Interestingly, NP vaccinated aged mice did produce significantly higher amounts of anti-NP IgG serum antibodies when compared to controls (Figure 3B). This still, however, was significantly lower than the amount of antibody produced by the vaccinated young mice. Furthermore, NP vaccination of young, but not aged mice, also reduced the level of albumin in the BAL when compared to the PBS control groups, indicating that lung damage was also reduced in the young but not aged lungs (Figure 3C).

We next examined G-CSF, IL-6 and IL-1α in BAL following flu infection (Figure 3D). The levels of these cytokines in BAL from naïve uninfected young and aged mice is shown in the “none” groups. In young NP vaccinated and infected mice (NP/PR8), levels of these cytokines were reduced significantly compared to PBS/PR8 control group, which was vaccinated with PBS and then received influenza infection. In contrast, aged vaccinated and infected mice (NP/PR8) showed no significant reduction in these cytokines when compared to the PBS/PR8 control group. Other cytokines and chemokines also exhibited a similar pattern of expression in young and aged groups (Sup Figure 3). We next examined cells from the BAL fluid in each of these groups and found that NP vaccination of both young and aged mice prior to influenza infection resulted in an enhanced percentage of neutrophils when compared to the PBS/PR8 control groups (Figure 3E). Thus, while NP vaccination generates a significant amount of NP-specific IgG in aged mice and protects from death due to influenza infection, it has no impact on viral load, BAL cytokines and chemokines or albumin levels.

### The role of antibody

**Figure 4:**
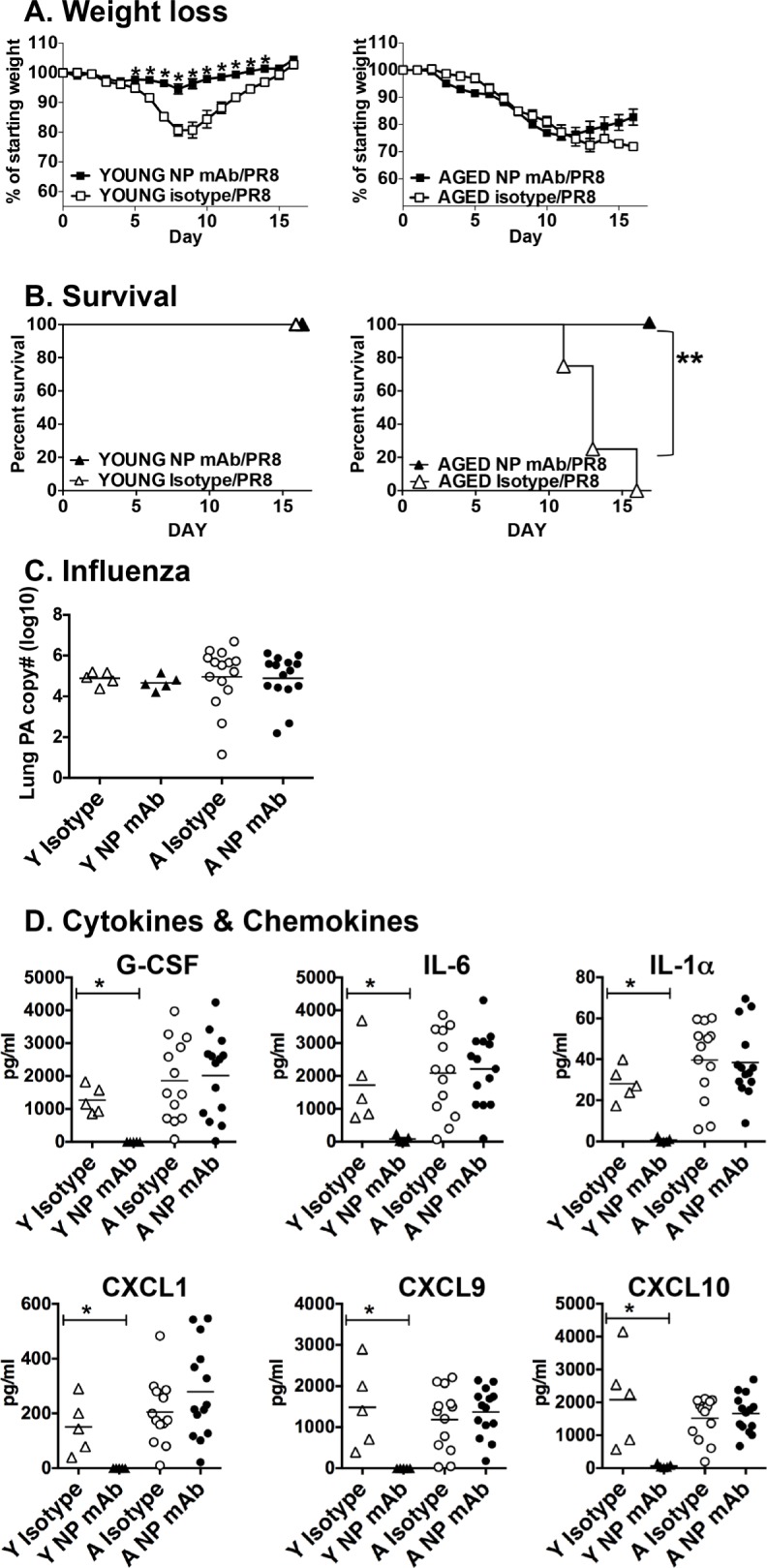
Impact of NP mAb transfer on influenza infection Young (2-3 mos.) and aged (19-22 mos.) C57BL/6 mice were administered anti-NP mAb or isotype control mAb (350 ug i.p.) on days -1 and day 0. On day 0, they were infected intranasally with 400 EID_50_ of PR8 influenza. **A.** Weight loss and **B.** survival were monitored following infection. **C.** On 5 dpi infection, mRNA was prepared from lung tissue from young and aged mice to quantitate influenza PA by real-time PCR. **D.** BAL was also harvested from young and aged mice on day 5 and cytokine and chemokine levels were determined by multiplex assay. *p < 0.05 by Student's *t* test comparing vaccinated and control groups; **p < 0.05 by Log rank test.

Since the aged mice do produce a significant amount of anti-NP IgG, we next sought to determine if the protective effect (survival from infection) of NP vaccination could be attributable to this antibody. To do this, we employed adoptive transfer of a NP-specific monoclonal antibody (mAb). We chose this approach since we have previously shown that this mAb protects young mice from secondary bacterial infection following influenza and reduces inflammatory mediators in the lungs of young influenza infected mice [18]. The NP mAb or isotype control was administered on days -1 and 0 of influenza challenge. Young mice receiving the NP mAb were protected from weight loss during influenza infection, while aged mice were not (Figure 4A). Importantly, aged mice that received the NP mAb were protected from death due to influenza infection (Figure 4B), much like those vaccinated with NP protein (Figure 2B). Treatment with the NP mAb had no impact on the amount of virus in young or aged groups at this time point (Figure 4C). In contrast, treatment of young mice with the NP mAb did significantly reduce the levels of BAL cytokines and chemokines but had no effect on the aged mice. (Figure 4D). Thus in our aged groups, survival from influenza-induced death and reduction in lung inflammation seem to be mediated separately, with Ab involved in survival but not in reducing lung inflammation.

### NP-specific T cells in vaccinated mice

**Figure 5:**
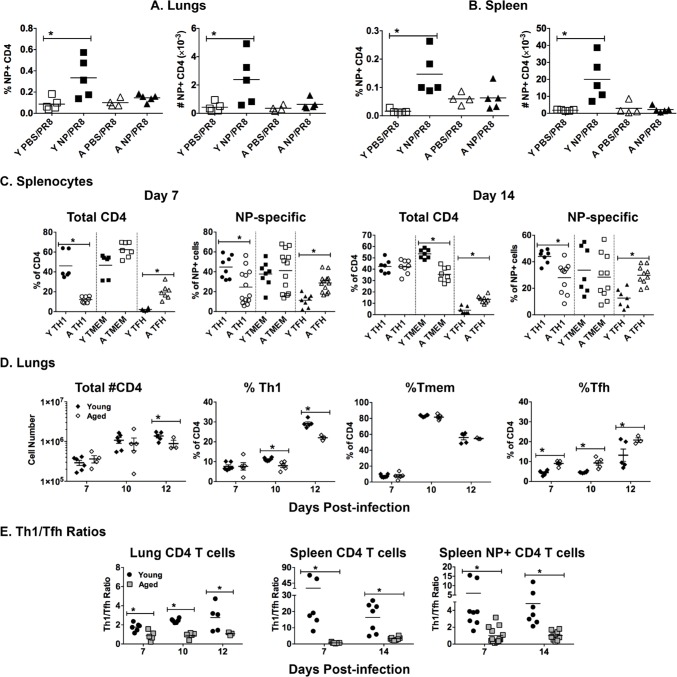
Age-related differences in T cells during influenza infection Young [Y] (2-3 mos.) and aged [A] (19-22 mos.) C57BL/6 mice were vaccinated with NP or PBS on days -31 and -21 and then infected with intranasally with 400 EID_50_ of PR8 influenza on day 0. On 5 dpi lungs and spleens were harvested. NP-specific CD4 T cells in the **A.** lungs and **B.** spleen were enumerated with a NP_311-325_/IA^b^ MHC Class II tetramer. **C.** On day 0, young and aged unvaccinated mice were infected intranasally with 400 EID_50_ of PR8 influenza. On 7 and 14 dpi, the CD4 T cell population was further broken down phenotypically into subsets based on PSGL1 (CD162) and Ly6C expression to indicate T helper type 1 (Th1, PSGL1^hi^ Ly6C^hi^), T memory (Tmem, PSGL1^hi^ Ly6C^lo^) and T follicular helper (Tfh, PSGL1^lo^ Ly6C^lo^) according to the scheme developed by Marshall and colleagues [38]. The percent of each subset within the total CD4 and NP-specific CD4 T cell populations is shown. **D.** On day 0, young and aged unvaccinated mice were infected intranasally with 400 EID_50_ of PR8 influenza. On 7, 10 and 12 dpi, lungs were harvested to generate single cell suspensions, which were then stained as in **C.**. Total CD4 numbers and percentages within Th1, Tmem and Tfh subpopulations are shown. **E.** Ratio of Th1/Tfh percentages from results shown in **C.** and **D.**. For all, data is representative of 2 independent experiments and each symbol represents a single animal; line shows the mean; *p < 0.05 by 2 way ANOVA with Bonferroni posthoc correction for A, B and D or Student's *t* test comparing young *vs* aged groups for C and E

Finally, we examined T cells in our vaccinated, influenza-infected young and aged groups from Figure 3. We focused on CD4 T cells since, like most subunit vaccines, our recombinant NP vaccination induces a mainly CD4 T cell response. NP-specific CD4 T cells were enumerated using a MHC Class II tetramer 5 dpi. In lungs (Figure 5A) and spleen (Figure 5B), there was a significantly higher percentage and number of NP-specific CD4 T cells in the young NP vaccinated groups (NP/PR8), compared to the control groups (PBS/PR8). In contrast, NP vaccinated cohorts of aged mice did not show any significant enhancement of CD4 T cell numbers in either spleen or lungs when compared to controls. Importantly, it is essential for these vaccine generated CD4 T cells to be recruited to the lungs during influenza infection in order to quickly and completely clear virus and resolve inflammation [31] and the lack of NP-specific T cells in the aged lungs could be a major contributor to slower resolution of infection.

While the effect of aging on CD8 T cell responses to influenza has been extensively studied [32-36], the impact on CD4 T cells is less well understood. Importantly, Th1 cells are an integral part of the immune response to influenza and are crucial to complete resolution of the infection [32, 37]. To begin to investigate the impact of aging on CD4 T cells during influenza infection, we examined the distribution of Th subsets in unvaccinated young and aged mice using a scheme developed in the Kaech laboratory [38]. The NP-specific CD4 T cell population was broken down phenotypically into subsets based on PSGL1 (CD162) and Ly6C expression to indicate T helper type 1 (Th1, PSGL1^hi^ Ly6C^hi^), T memory (Tmem, PSGL1^hi^ Ly6C^lo^) and T follicular helper (Tfh, PSGL1^lo^ Ly6C^lo^). Representative flow cytometery plots of PSGL1 and Ly6C stained splenic NP-specific CD4 T cells from young and aged influenza infected mice are shown in Supp Figure 4A. The percentage (Figure 5C) and numerical (Sup Figure 4B) distribution of these Th subsets within the spleens of influenza infected mice show that on both 7 and 14 dpi within the total CD4 population and the NP-specific CD4 population, skewing towards Th1 is more prevalent in the young groups. Furthermore, Figure 5C also demonstrates that the percentage of Tfh phenotype CD4 T cells in both the total CD4 and NP-specific populations was significantly greater in the aged groups. This observation also holds up when we use a more conventional phenotypic analysis of splenic Tfh cells. As shown in Supp Figure 4C, CXCR5+PD-1+ Tfh percentages and total numbers are higher in aged groups both prior to and during influenza infection. Thus, with aging we have found a decrease in Th1 and an increase in Tfh differentiation.

Interestingly, the total number of CD4 T cells in the lungs at 7 and 10 dpi is not different between young and aged and is only significantly different on 12 dpi (Figure 5D). In the lungs during influenza infection we also find a significant skewing of the total CD4 population towards Th1 in the young and towards Tfh in the aged at all time points examined. To further assess this subset differentiation, we compared the Th1/Tfh ratio of the results presented in Figure 5C and 5D. This comparison (Figure 5E) demonstrates that during influenza infection in both the lungs and spleen Th1 differentiation dominates significantly in young groups, while Tfh differentiation dominates in the aged. This holds true for both the total CD4 population as well as the NP+ tetramer population and for all time points examined. Thus, while the total number of CD4 T cells trafficking to the lungs in unvaccinated influenza infected aged mice is not significantly different, the Th subset distribution of these cells is dramatically altered and could have an impact on protective immunity.

## DISCUSSION

With age, the immune response to influenza infection diminishes resulting in slower viral clearance and increased lung inflammation. In older individuals, the result is exacerbation of chronic age-related diseases and increased susceptibility to secondary bacterial infections, which are often fatal [39]. We also observed this in our aging mouse model of influenza infection. Aged mice were slower to clear virus and exhibited higher levels and extended production of inflammatory cytokines and chemokines in BAL fluid when compared to young. Furthermore, other cytokines and chemokines important for recruitment of an adaptive immune response to influenza infection in the lungs were either delayed or produced at significantly lower levels in aged mice. Thus, while inflammation is lingering in response to infection, recruitment of virus-specific T cells is delayed due to lower levels of lung chemoattractants. Interestingly, there was not a dramatic difference in either the percentage, number or type of cells in the BAL of aged mice during influenza infection. This observation indicates that there may be an increase in the amount of cytokine produced per cell in aged lungs, which may be attributed to the higher viral burden when compared to young. Thus, ways to better control viral levels, especially in older individuals, are an important clinical goal. One of the easiest ways to prevent influenza-related complications is by vaccination and our previous work has shown that vaccination can prevent lung inflammation following influenza infection in young mice [18]. In this study we have gone on to examine the protection induced by NP vaccination in aged mice.

It is well documented that the efficacy of influenza vaccination declines with aging [40, 41] but the exact mechanisms responsible for this remain elusive. Here, we have examined the efficacy of an influenza vaccine consisting of the highly conserved influenza NP protein. This vaccine was chosen because it has been shown to induce a protective heterosubtypic antibody response in young mice [42, 43] and can also reduce lung inflammation and susceptibility to secondary bacterial infection following a primary influenza infection [18]. In our current study, NP vaccination of aged mice did protect from death following influenza infection but did not protect from influenza-induced weight loss. In addition, while vaccination did protect young mice from death due to secondary bacterial infection, this was not the case in the aged cohorts, where significant death was observed. This could be attributable to the fact that NP vaccination significantly reduced the levels of inflammatory cytokines and chemokines and albumin, a marker of lung damage, in young but not aged lungs following influenza infection. In fact, it has been suggested that damage to the lung epithelium during influenza infection facilitates bacterial colonization and is responsible for the enhanced susceptibility to bacterial infection [44]. In contrast, NP vaccination did induce a significant amount of NP-specific IgG in aged mice, although this amount was less than what was generated in young mice. Adoptive transfer of a NP-specific monoclonal antibody to aged influenza-infected mice had protective capacity similar to NP vaccination, indicating that some of the protective capacity of the vaccination may be working *via* an antibody-dependent mechanism.

In addition to production of NP-specific antibodies, young, but not aged, NP vaccinated mice given a subsequent influenza infection also exhibit NP-specific CD4 T cells in the lungs and spleen very early (day 5) during infection. CD4 T cell effectors in the lungs have been shown to be extremely important in viral clearance and can be cytotoxic toward virally infected cells [45-47]. Thus, it is likely that the presence of these CD4 T cells is helping to dampen inflammatory mediators in the young lungs during infection, especially since these mice have very low levels of albumin in their lungs indicating minimal lung damage. These results are very interesting since a recent study from the Blackman laboratory demonstrated that there is a significant delay in influenza-specific Th1 effector CD4 T cell appearance in the lungs during infection [48]. So it seems that while the CD4 T cell response needed to generate an antibody response in our aging model is adequate, the ability to generate lung-homing Th1 effectors during influenza infection is less preserved with aging. This could account for the reduced ability to control lung inflammatory mediators that we observed in our study.

Importantly, our results point to a novel finding that the two mechanisms of vaccine-mediated protection, antibody and lung-homing T cell effector generation, seem to be differentially affected by aging. In order to understand what is happening in our aging model, we went on to examine the CD4 T cells of influenza-infected mice and interrogate the impact of age on Th subset differentiation. We used a Th subset identification protocol developed in the Kaech laboratory to distinguish Th1, T memory and Tfh subsets [38]. Interestingly, we found that in both the total CD4 T cell population and the influenza NP-specific CD4 T cell population there was a significant skewing towards a Tfh phenotype in aged groups. In contrast, young CD4 T cells exhibited a skewing towards a Th1 phenotype, which is important for control of virus and inflammation in the lungs. This is seen most clearly when we compare the Th1 to Tfh ratios as demonstrated in Figure 5E, where most of the ratios from aged cohorts are less than 2 and those from young cohorts range to over 50. Our results confirm those of Sage and colleagues [49] who also reported similar enhancement of the Tfh population in aged mice. This difference in Th subset distribution between young and aged groups could account for the fact that aging has a greater impact on the generation of lung-homing Th1 effectors rather than on Tfh cells and antibody production. Whether this predilection for Tfh differentiation is intrinsic to aged CD4 T cells or is induced by the aged microenvironment is yet to be determined. Nonetheless, this knowledge of differences in Th subset distribution with aging could help further our understanding of how aging impacts immunity to both infections and vaccinations. These results could also be useful for choosing adjuvants for vaccines for elderly populations. A recent study demonstrated that vaccine adjuvants are extremely important for influencing the direction of an immune response [50] and that more novel adjuvants such as GLA-SE and IC31 are much better at driving a Th1-like response. Finally, our results also stress why it is important to examine multiple parameters, such as both humoral and cellular responses, when testing new vaccines and adjuvants since each of these can be differentially influenced by aging.

## MATERIALS AND METHODS

### Mice

Young (2-3 months) C57BL/6 mice were bred in the specific pathogen free Trudeau Institute Animal Breeding Facility or purchased from Jackson Laboratories. Aged (19-22 months) C57BL/6 mice were obtained from the National Institute on Aging rodent colony. All mice were cared for in accordance with the recommendations in the Guide for the Care and Use of Laboratory Animals of the National Institutes of Health. All procedures were approved by the Trudeau Institutional Animal Care and Use Committee (IACUC), protocol number 02-021 or by the University of Connecticut Medical School IACUC, protocol number 100705. Recumbent mice and mice that lost more than 30% body weight were considered moribund and euthanized.

#### Viral infection

To infect with Influenza virus A/PR/8/34 (PR8), 400 EID_50_ were given intranasally in 40μl to isoflurane anesthetized animals. At the indicated time points, the viral burden in mRNA from digested whole lung tissue was determined by real-time PCR measuring viral acid polymerase (PA) copy number [25].

#### Multiplex

Bronchiolar lavage fluid (BAL) was collected by flushing lungs with 1ml saline. Supernatant was collected after centrifugation and assayed for cytokine and chemokine content using the Luminex Mouse Cytokine/Chemokine 32-plex panel (Millipore).

#### Albumin assay

The albumin content in BAL supernatant was determined by ELISA (Bethyl Laboratories).

#### Differential counts

BAL cells were centrifuged, resuspended in 100ul PBS and cytospin slides were generated. After drying, differential stain (Diff-quick, IMEB Inc.) was applied according to manufacturer's protocol. Differential counts (200 cells per sample) were performed to distinguish the percent macrophages, neutrophils and lymphocytes.

#### Antibody assay

Anti-NP IgG titers in serum were determined by ELISA using HRP-labeled anti-isotype antibodies (Southern Biotech Inc). Antibody titers were determined by the last serum dilution with an optical density above background.

### Vaccination

Recombinant A/PR/8/34 influenza nucleoprotein (NP) was generated as a C terminal histidine-tagged protein in *E. coli* and isolated using the ProBond system (Invitrogen), as described [42]. Intraperitoneal immunizations contained 30 μg NP in alum with 20 μg *E. coli* serotype 0111:B4 lipopolysaccharide (LPS; Enzo Life Sciences) and were carried out at 30 and 20 days prior to influenza infection. Control groups received saline in alum with LPS.

### Bacterial infection

Serotype 4 *Streptococcus pneumoniae* (ATCC strain 6304) grown on blood agar plates was used to inoculate Tryptic soy broth cultures, which were grown at 37°C without shaking in sealed tubes. After dilution to an OD_600nm_ of 0.15, they were re-grown to an OD_600nm_ of 0.45, washed with saline, and approximately 250 CFU were applied in a volume of 50 μl saline to the nares of anesthetized mice. The number of bacteria in the inoculating dose was confirmed by plating. The intranasal median lethal dose of strain 6304 is approximately 1.5×10^4^ CFU when grown as described above and administered to naïve mice.

### Flow cytometry

Single cell suspensions were generated from spleens, lymph nodes and lungs of young and aged mice. Cells were incubated with Fc block (anti-CD16/32) for 15 min on ice followed by staining with NP_311-325_IA^b^ MHC Class II tetramer (generated by the Trudeau Institute Molecular Biology Core [http://www.trudeauinstitute.org]) for 1 h at room temperature. This was followed by staining with anti-CD4 (eBioscience) and anti-Ly6C and anti-PSGL-1 (eBioscience). Samples were acquired on LSR II flow cytometer (BD Biosciences), and data were analyzed with FlowJo software (Tree Star).

### Antibody treatment

Passive transfer of mouse IgG2a NP-specific mAb H16-L10-4R5/HB-65 was achieved by administering 350 μg intraperitoneal injections on the day of and the day prior to PR8 influenza challenge. Control mice received isotype matched mAb C1.18.4. All mAb were Protein G purified and supplied by BioXcell, who reported < 2 endotoxin units per mg.

### Statistics

Survival curves were analyzed by Log rank tests. In all other studies, differences between vaccinated and unvaccinated groups or between young and aged groups were analyzed by Student's *t* test or 2 or 3 way ANOVA with Bonferroni posthoc correction.

## SUPPLEMENTARY FIGURES


