# Supplementary material for: Vaccine efficacy and T helper cell differentiation change with aging
Source: Oncotarget. 2016 May 9;7(23):33581–94. doi: 10.18632/oncotarget.9254 (PMC5085104; doi:10.18632/oncotarget.9254)
Supplement: Supplementary file 1 [file oncotarget-07-33581-s001.pdf]

Vaccine efficacy and T helper cell differentiation change with aging

Supplementray Material

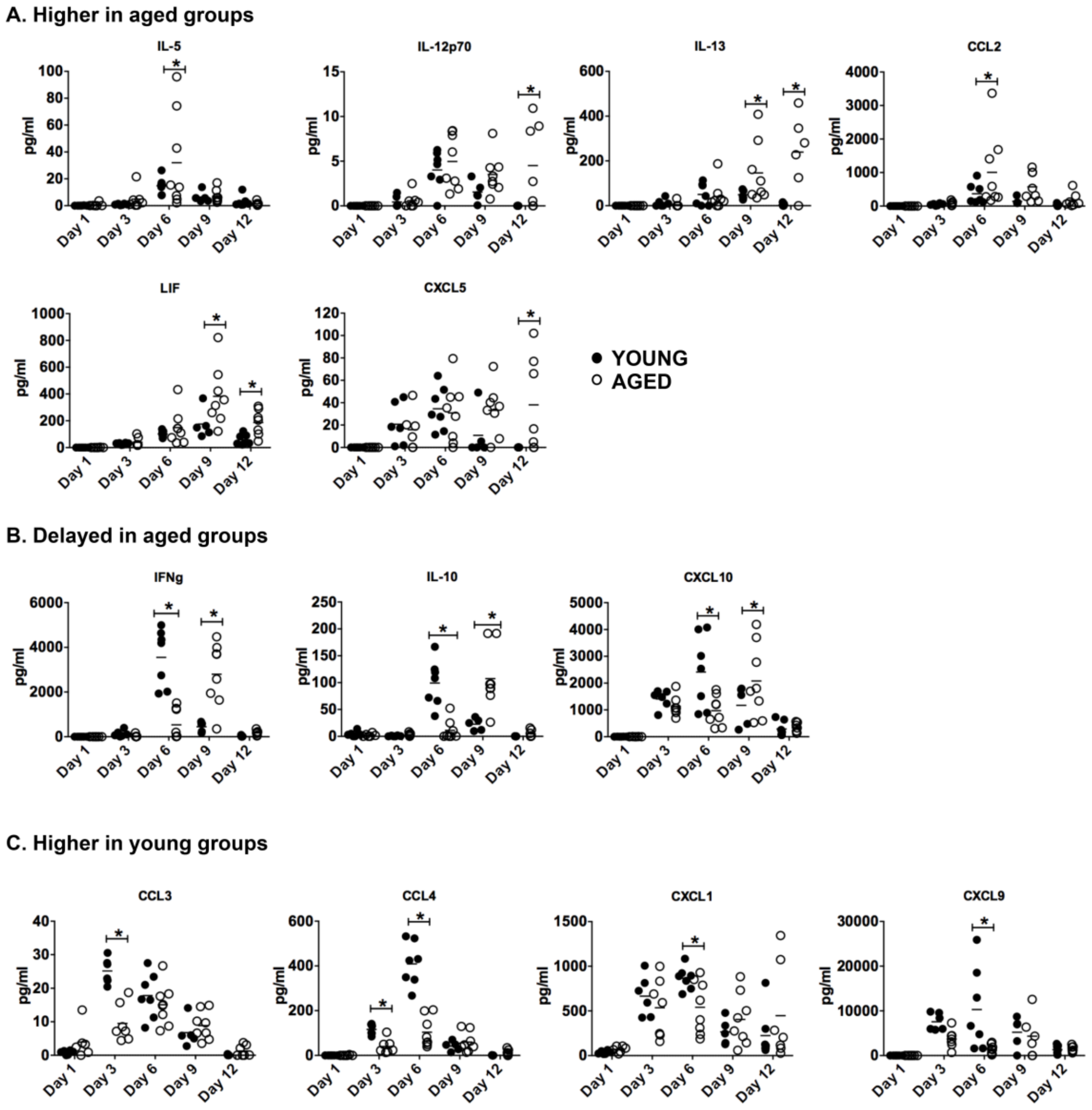

SUPPLEMENTARY FIG 1. Additional cytokines and chemokines measured by multiplex assay of samples in Fig 1C.

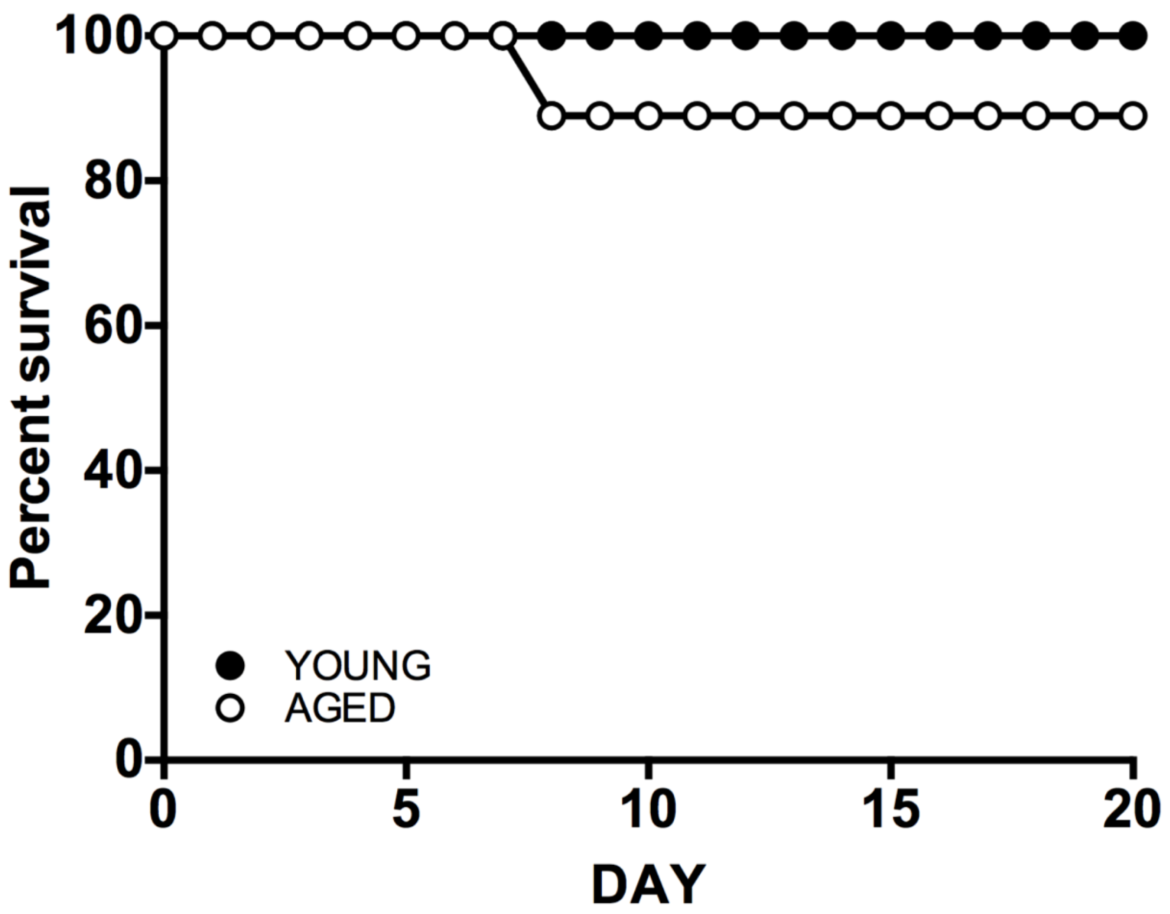

SUPPLEMENTARY FIG 2. Survival of young and aged mice given 250 CFU of *Streptococcus pneumoniae.*

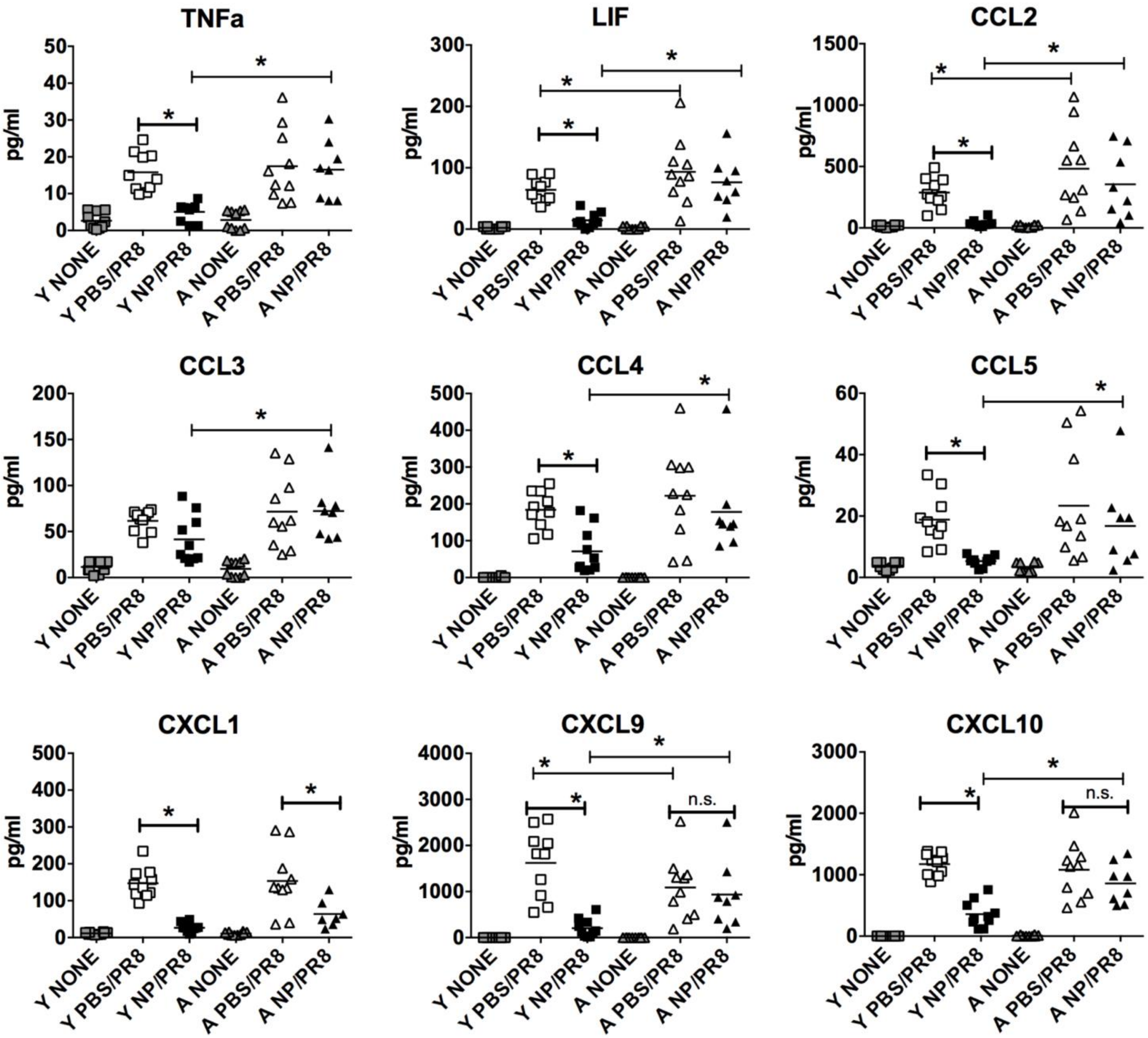

SUPPLEMENTARY FIG 3. Additional cytokines and chemokines measured by multiplex assay of samples in Fig 3D.

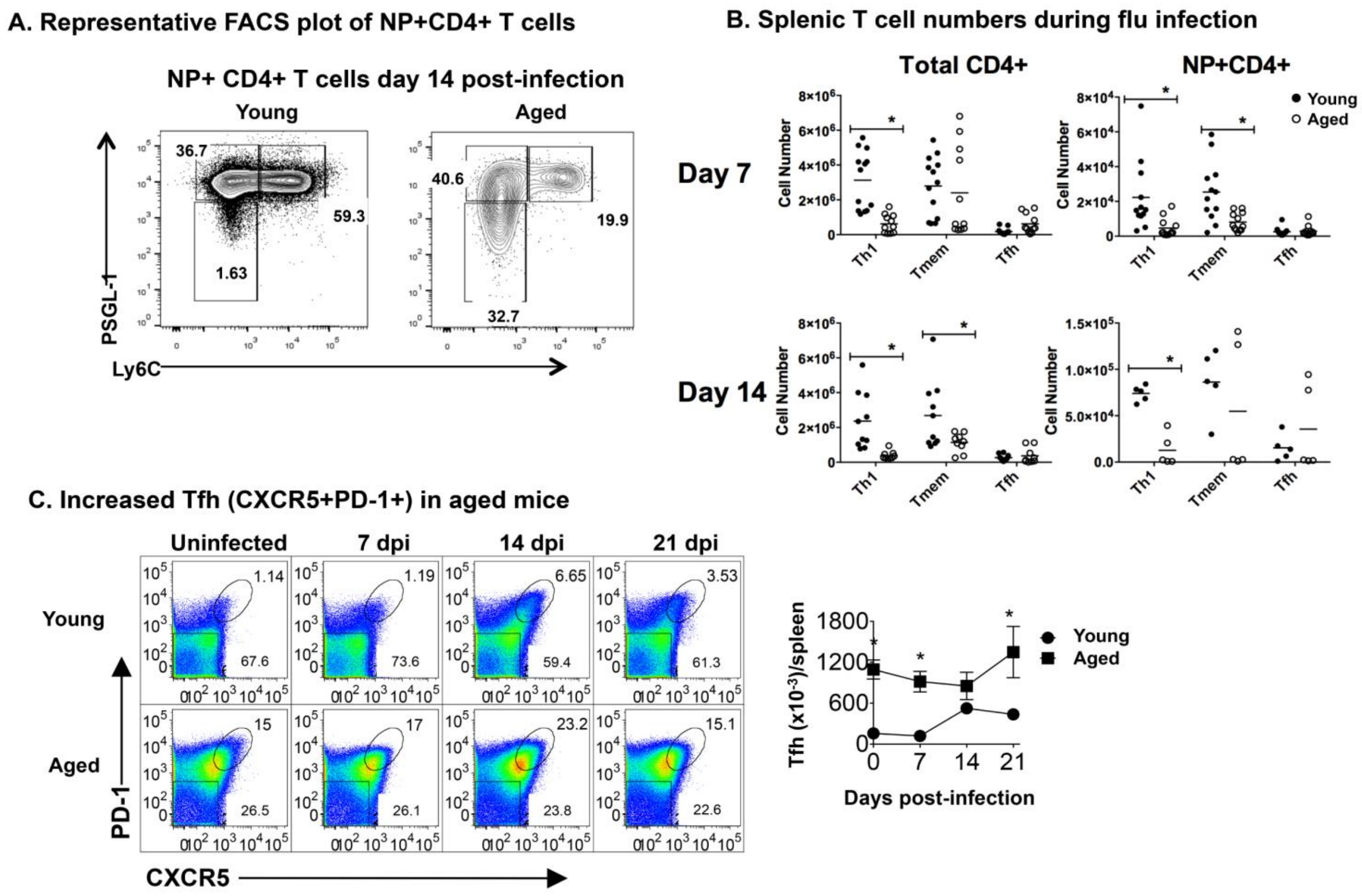

SUPPLEMENTARY FIG 4. Additional information on CD4 T cells in Figure 5. (A) Representative FACS plot of splenic NP+CD4 T cells on day 14 of influenza infection. (B) Cell numbers of graphs presented in Figure 5C. (C) Kinetic analysis of Tfh (CXCR5+PD-1+) within the total CD4 T cell population in spleens of young and aged mice during influenza infection. Representative FACS dot plots and graphical representation of cell numbers are shown.
